# Identification of seven tumor‐educated platelets RNAs for cancer diagnosis

**DOI:** 10.1002/jcla.23791

**Published:** 2021-05-06

**Authors:** Xinxin Ge, Liuxia Yuan, Bin Cheng, Kesheng Dai

**Affiliations:** ^1^ The First Affiliated Hospital and Collaborative Innovation Center of Hematology Jiangsu Institute of Hematology Cyrus Tang Medical Institute State Key Laboratory of Radiation Medicine and Protection Key Laboratory of Thrombosis and Hemostasis Ministry of Health National Clinical Research Center for Hematological Diseases Soochow University Suzhou China

**Keywords:** bioinformatics analysis, diagnosis, mRNA, tumor educated platelets

## Abstract

**Background:**

Tumor‐educated platelets (TEPs) may enable blood‐based cancer diagnosis. This study aimed to identify diagnostic TEPs genes involved in carcinogenesis.

**Materials and Methods:**

The TEPs differentially expressed genes (DEGs) between healthy samples and early/advanced cancer samples were obtained using bioinformatics. Gene ontology (GO) analysis and Kyoto encyclopedia of genes and genomes (KEGG) pathway enrichment analysis were used to identify the pathways and functional annotation of TEPs DEGs. Protein‐protein interaction of these TEPs DEGs was analyzed based on the STRING database and visualized by Cytoscape software. The correlation analysis and diagnostic analysis were performed to evaluate the diagnostic value of TEPs mRNAs expression for early/advanced cancers. Quantitative real‐time PCR (qRT‐PCR) was applied to validate the role of DEGs in cancers.

**Results:**

TEPs mRNAs were mostly involved in protein binding, extracellular matrix, and cellular protein metabolic process. RSL24D1 was negatively correlated to early‐stage cancers compared to healthy controls and may be potentially used for early cancer diagnosis. In addition, HPSE, IFI27, LGALS3BP, CRYM, HBD, COL6A3, LAMB2, and IFITM3 showed an upward trend in the expression from early to advanced cancer stages. Moreover, ARL2, FCGR2A, and KLHDC8B were positively associated with advanced, metastatic cancers compared to healthy controls. Among the 12 selected DEGs, the expression of 7 DEGs, including RSL24D1, IFI27, CRYM, HBD, IFITM3, FCGR2A, and KLHDC8B, were verified by the qRT‐PCR method.

**Conclusion:**

This study suggests that the 7‐gene TEPs liquid‐biopsy biomarkers may be used for cancer diagnosis and monitoring.

## Graphical abstract

Receiving operating characteristic (ROC) curves show the relative diagnostic performance of TEPs RSL24D1, HPSE, IFI27, LGALS3BP, CRYM, HBD, COL6A3, LAMB2, IFITM3, ARL2, FCGR2A, and KLHDC8B have predictive validation value for cancers.

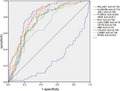

## 1 INTRODUCTION

Cancer is one of the leading causes of mortality worldwide.^1^ Tumor tissue biopsy is considered as a gold standard for cancer subtyping. Yet, it is an expensive, time‐consuming, and invasive technique. In addition, samples obtained by standard biopsy may be insufficient to reflect the entire tumor's characteristics.^2^

Recently, non‐invasive liquid biopsies have become a promising alternative for tumor diagnosis, prediction, and monitoring, especially for solid tumors that cannot be obtained by conventional tissue biopsy.^3^ Compared to traditional tissue biopsy, liquid biopsies offer the following advantages: (1) non‐invasiveness; (2) few side effects and repeatability; (3) do not rely on imaging examinations; (4) effectivity in dealing with tumor heterogeneity.

To date, many blood‐based biomarkers, including circulating nucleic acids, circulating tumor cells (CTCs), extracellular vesicles, and TEPs have been used for cancer detection and diagnosis.^4^, ^5^, ^6^, ^7^, ^8^, ^9^, ^10^ Nevertheless, CTCs and ctDNA detection is difficult and not equally feasible in all tumor entities.^11^ As a second most abundant cell type in peripheral blood generated from megakaryocytes' cytoplasm, platelets are a central regulator of thrombosis and hemostasis.^12^ Platelets can ingest cellular RNA and proteins.^13^, ^14^ Recent studies have demonstrated that platelets have essential roles in tumor progression and metastasis.^15^, ^16^ They can be directly or indirectly activated by tumor cells, leading to their behavior and RNA profile alteration.^17^, ^18^

Tumor‐educated platelets (TEPs) can promote tumor metastasis.^15^, ^19^, ^20^ TEPs RNAs have been emerging as potential blood‐based biomarkers for cancer diagnosis, prognosis, and prediction.^9^, ^10^ Recent studies have proved that TIMP1 and TGA2B mRNA in TEPs and a three‐platelet mRNA set (MAX, MTURN, and HLA‐B) may be used as a diagnostic biomarker for colorectal cancer and lung cancer.^21^, ^22^, ^23^, ^24^ Yet, the role and function of TEP mRNAs in other types of cancers are still not clear. Most platelet profile analysis data from patients with different cancer have been uploaded to public databases, but thorough analysis has not been performed. Data from independent studies are also limited by their sources (i.e., single cohort studies) and sample heterogeneity. Therefore, the use of integrated bioinformatics methods to re‐analyze these data may provide new insights for further cancer diagnosis.

## 2 MATERIAL AND METHODS

### 2.1 Microarray data

Based on the inclusion criteria, two gene expression profiles (GSE68086 and GSE89843) were downloaded. The array data GSE68086 includes 285 platelet samples covering 55 healthy samples, 39 early localized cancer samples, and 191 advanced metastatic cancer samples. The GSE89843 database includes 636 samples covering 234 healthy samples, 55 early‐localized cancer samples, and 345 metastatic cancer samples. In their study, advanced, metastatic cancer or metastatic cancer patients were defined as cancer patients with clear metastasis from the primary tumor to distant organ(s).^9^, ^10^

### 2.2 Data process

EdgeR package was used to identify the differential expression of genes by linear modeling. Genes with FC (fold change) > 1 and adj *p*‐value (adjusted *p*‐value) < 0.05 were considered to be differentially expressed in platelets collected from early/metastatic cancer samples and healthy samples. R software was then quoted to obtain the heatmaps and volcano plots about differential expression of genes in TEPs.

### 2.3 Functional and pathway enrichment analysis

Gene ontology (GO) analysis was used to reveal the function of genes and gene products in many organisms.^25^ Kyoto Encyclopedia of Genes and Genomes (KEGG) allots genes and genomes functional meanings at the molecular and higher levels.^26^ DAVID (Database for Annotation Visualization and Integrated Discovery, http://www.david.niaid.nih.gov) is a database that is applied for annotation, visualization, and integrated discovery.^27^ *p*‐value < 0.1^28^ was considered as enriched.

### 2.4 PPI network construction

The Search Tool for the Retrieval of Interacting Genes (STRING, https://string‐db.org/) database aims to collect and integrate the information, which represents all functional interactions between the expressed proteins through strengthening known and predicted protein‐protein association data among plenty of organisms.^29^ Thus, the protein‐protein interaction (PPI) network of DEGs was built using a STRING database. The Molecular Complex Detection (MCODE) based on Cytoscape was applied to screen modules of the PPI network with degree cutoff = 2, node score cutoff = 0.2, k‐core = 2.

### 2.5 Patients and healthy volunteers

Approval for obtaining whole‐blood samples from healthy volunteers and patients was obtained from the Ethics Committee of The First Affiliated Hospital of Soochow University (NO: 2017065). 19 CRC patients, 16 NSCLC patients, and four healthy volunteers were recruited for the studies. The details are described in Table S8.

### 2.6 Washed platelet preparation, RNA extraction, and quantitative real‐time PCR

Washed platelets were prepared from whole blood. All procedures were done at room temperature. PRP was separated by centrifugation of blood at 200 *g* for 8 min. Platelets were washed with CGS buffer (0.123 M NaCl, 0.033 M d‐glucose, 0.013 M trisodium citrate, pH 6.5). Total RNA was extracted from washed platelet from patients or healthy volunteers using TRIzol reagent (15596026, Thermo Fisher Scientific), and the RNA concentration was determined using a NanoDrop‐2000 Spectrophotometer (Thermo Fisher Scientific). Then, the RNA was reverse transcribed to cDNA using RevertAid First Strand cDNA synthesis kit (k1622, Thermo Fisher Scientific) according to the manufacturer's instruction. Quantitative real‐time PCR (qRT‐PCR) was performed using primers specific for RSL24D1, HPSE, IFI27, LGALS3BP, CRYM, HBD, COL6A3, LAMB2, IFITM3, ARL2, FCGR2A, KLHDC8B, and GAPDH on a LightCycler 96 instrument (Roche, Indianapolis, IN, USA). Primer sequences were as follows:

KLHDC8B‐ forward, 5′‐GACACTGCTGAGACACTGGACATG‐3′;
KLHDC8B‐ reverse, 5′‐TCATCCACACCACCCACCACTAG‐3′;
FCGR2A‐ forward, 5′‐CCAGGAGGACTCTGTGACTCTGAC‐3′;
FCGR2A‐ reverse, 5′‐GCTGCGTGTGGGTGGGAATG‐3′;
ARL2‐ forward, 5′‐GAACTACTTTGAGAGCACCGAT‐3′;
ARL2‐ reverse, 5′‐ATTAGCAAAGATGAGGAGGGTT‐3′;
IFITM3‐ forward, 5′‐CTTCTTCTCTCCTGTCAACAGT‐3′;
IFITM3‐ reverse, 5′‐GTTCATGAAGAGGGTGTTGAAC‐3′;
LAMB2‐ forward, 5′‐TGGTGCTAGAGATGTTTAGTGG‐3′;
LAMB2‐ reverse, 5′‐TCAGAACTCAGTGAACCTTGAG‐3′;
COL6A3‐ forward, 5′‐CATGTTCTCCTTGGACACCTAC‐3′;
COL6A3‐ reverse, 5′‐GTAGCGAATCTCGTCACTAGAA‐3′;
HBD‐ forward, 5′‐AGAGGTTCTTTGAGTCCTTTGG‐3′;
HBD‐ reverse, 5′‐CTCACTCAGCTGAGAAAAAGTG‐3′;
CRYM‐ forward, 5′‐TGCCCTGAAGGAGTCTGGAGATG‐3′;
CRYM‐ reverse, 5′‐AACTGTGTCTTCCACTGCCATTCC‐3′;
LGALS3BP‐ forward, 5′‐CAATGGTACTTCTACTCCCGAA‐3′;
LGALS3BP‐ reverse, 5′‐GAACTGTAGGCAGAGCTTCTC‐3′;
IFI27‐ forward, 5′‐TCACTGGGAGCAACTGGACTCTC‐3′;
IFI27‐ reverse, 5′‐TCGCAATGACAGCCGCAATGG‐3′;
HPSE‐ forward, 5′‐AGTGGATGAAAACTTCGATCCT‐3′;
HPSE‐ reverse, 5′‐CAGTGTTTGTGCAATGAAGGTA‐3′;
RSL24D1‐ forward, 5′‐ CTGGTAAAGAGCTTACAGTGGA‐3′;
RSL24D1‐ reverse, 5′‐TCTTCAACTCTCTTCATCGCAT‐3′;
GAPDH‐ forward, 5′‐GGACCTGACCTGCCGTCTAG‐3′;
GAPDH ‐ reverse5′‐GTAGCCCAGGATGCCCTTGA‐3′.

### 2.7 Statistical analysis

SPSS version 23.0 software (SPSS, Chicago, IL, USA) and GraphPad Prism version 8 software was used to perform statistical analysis. Spearman correlation coefficient was used to assess the correlation between the average gene expression and that of the sample group for identifying genes whose expression change goes up or down strictly monotonically with respect to the group. The Mann–Whitney test was then applied to identify the differential expressed genes among the different stages. Receiving operating characteristic (ROC) curve analysis was used to evaluate the discriminatory power of the combinations. Data were shown as median ±interquartile range. Differences were considered significant at *p* < 0.05. Different levels of significance were indicated as *p < 0.05, **p < 0.01, and ***p < 0.001.

## 3 RESULTS

### 3.1 Analysis of the mRNA profiles of TEPs from localized or metastatic cancer patients as compared to platelets from healthy controls

**FIGURE 1:**
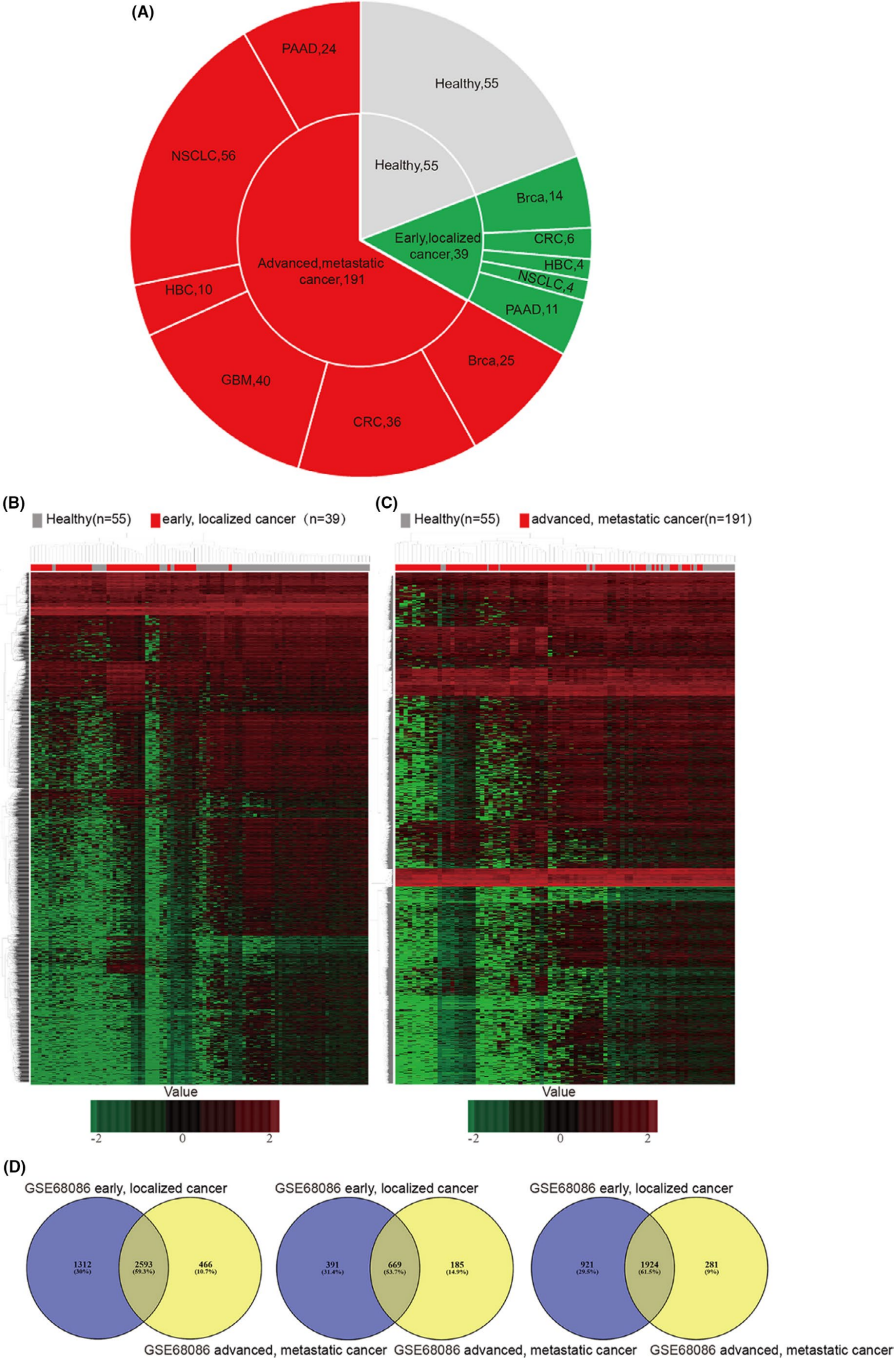
Identification of DEGs in TEPs in localized and metastatic pan‐cancer patients, and platelets from healthy controls, based on the datasets GSE68086. (A) A number of platelet samples of healthy controls and cancer patients with different stages or types of cancer‐based on GSE68086. (B, C) Hierarchical clustering heatmap of DEGs in the expression profiling datasets GSE68086. (B) Heatmap of DEGs in TEPs collected from healthy controls and early, localized cancer patients. (C) Heatmap of DEGs in TEPs collected from healthy controls and advanced metastatic cancer patients. The horizontal axis indicates the sample, and the vertical axis indicates the DEGs. Red represents the up‐regulated DEGs, and green represents the down‐regulated DEGs. DEGs, differentially expressed genes. (D) Identification of TEPs mRNAs between localized and metastatic pan‐cancer. Left, commonly altered differential expressed TEPs mRNAs. Middle, identification of up‐regulated differential expressed TEPs mRNAs. Right, identification of down‐regulated differential expressed TEPs mRNAs

The early, localized cancer patient cohort downloaded from the next‐generation sequencing data of GSE68086 included five tumor types containing breast cancer (BrCa, *n* = 14), colorectal cancer (CRC, *n* = 6), hepatobiliary cancer (HBC, *n* = 4), non‐small cell lung carcinoma (NSCLC, *n* = 4), and pancreatic cancer (PAAD, *n* = 11). The advanced, metastatic cancer patient cohort consisted of six tumor types, including BrCa (*n* = 25), CRC (*n* = 36), glioblastoma (GBM, *n* = 40), HBC (*n* = 10), NSCLC (*n* = 56), and PAAD (*n* = 24) (Figure 1A). There were 3905 differentially expressed mRNAs, among which 1060 were up‐regulated, and 2845 were down‐regulated in early‐ localized cancer‐treated platelets compared to platelet samples of healthy controls. Moreover, 3059 differentially expressed mRNAs were found, among which 854 were up‐regulated, and 2205 were down‐regulated in advanced, metastatic cancer treated platelets compared to platelet samples of healthy controls (Figure 1B,C, Figure S1A,B, Tables S1 and S2). To further investigate the differential expression among early‐ localized cancer‐treated platelets and metastatic cancer‐treated platelets, we explored commonly altered genes using Venny version 2.1.0 (https://bioinfogp.cnb.csic.es/tools/venny/index.html). We verified 2593 commonly altered differential mRNAs from the above platelet RNA‐sequencing datasets; among them, 669 were consistently up‐regulated, and 1924 were consistently down‐regulated (Figure 1D).

### 3.2 mRNA profiles of TEPs in localized and metastatic NSCLC cancer

**FIGURE 2:**
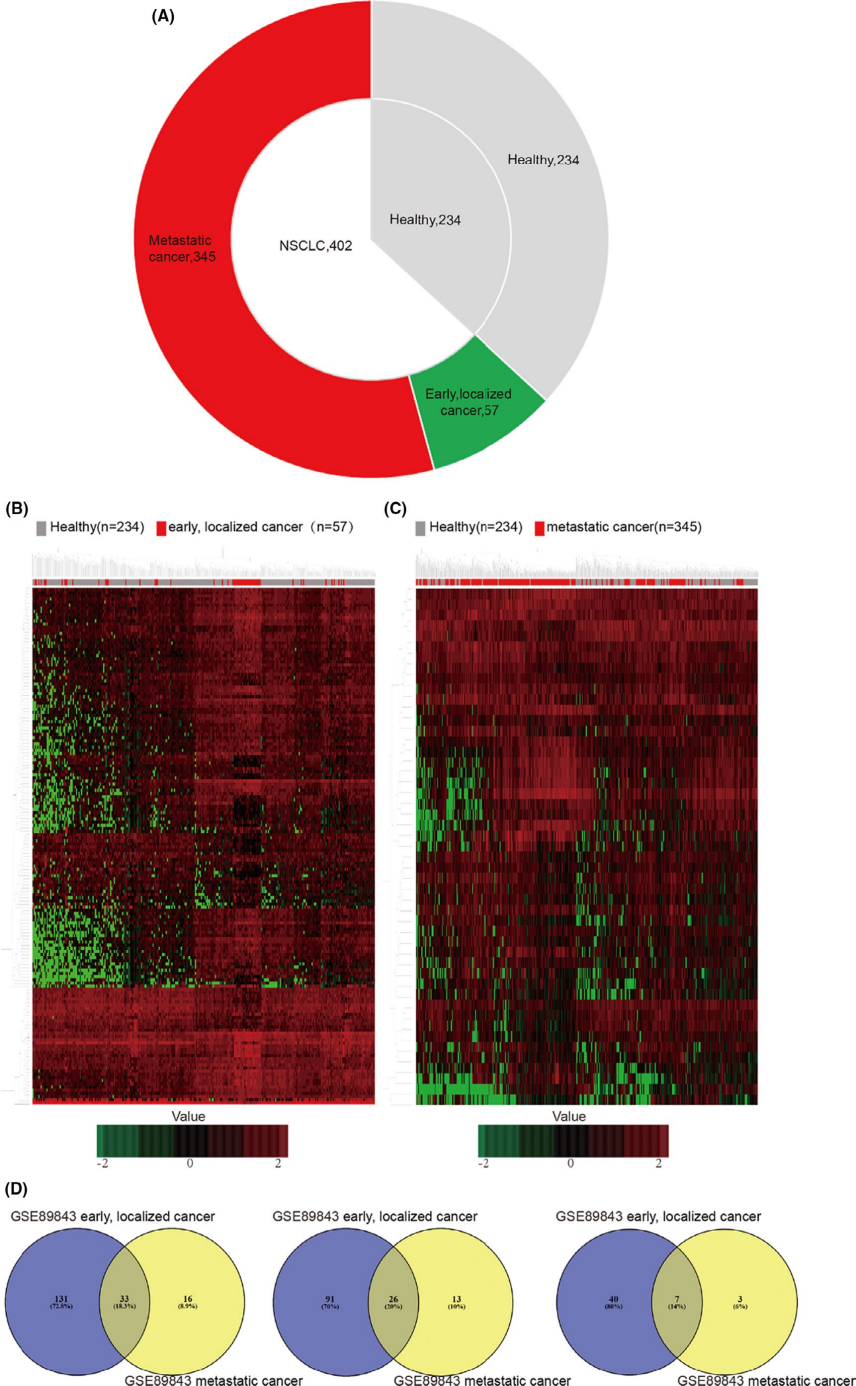
mRNA profiles of TEPs from localized and metastatic NSCLC cancer patients, and platelets from healthy controls, based on the datasets GSE89843. (A) Number of platelet samples of healthy controls and NSCLC cancer patients at different stages. (B, C) Hierarchical clustering heatmap of DEGs in the expression profiling datasets GSE89843. (B) Heatmap of DEGs in TEPs collected from healthy controls and early, localized NSCLC cancer patients. (C) Heatmap of DEGs in TEPs collected from healthy controls and metastatic NSCLC cancer patients. The horizontal axis indicates the sample, and the vertical axis indicates the DEGs. Red represents the up‐regulated DEGs, and green represents the down‐regulated DEGs. (D) Identification of TEPs mRNAs between localized and metastatic NSCLC cancer. Left, commonly altered differential expressed TEPs mRNAs. Middle, identification of up‐regulated differential expressed TEPs mRNAs. Right, identification of down‐regulated differential expressed TEPs mRNAs

The data of GSE89843 include 779 platelet samples collected from healthy controls (*n* = 234), individuals without reported cancers but with inflammatory conditions (*n* = 143), early‐localized (*n* = 57) or metastatic NSCLC cancer (*n* = 345), respectively. In this study, 636 samples were selected for further analysis, excluding individuals with inflammation (Figure 2A). Screening the differentially expressed genes, 164 differentially expressed mRNAs were identified between 57 early‐localized tumor‐educated platelet samples and 234 platelet samples from healthy controls; 117 were up‐regulated and 47 down‐regulated genes (Table S3). In addition, there were 49 differentially expressed mRNAs between 402 metastatic NSCLC treated platelet samples and 234 platelet samples from healthy controls, including 39 up‐regulated and 10 down‐regulated genes (Table S4).

Hierarchical clustering and a volcano plot were implemented to identify the differentially expressed mRNAs (Figure 2B,C, Figure S2A,B). As shown in Figure 2D, among the 33 commonly altered differential mRNAs from above platelets RNA‐sequencing datasets, 26 were consistently up‐regulated, and seven were consistently down‐regulated.

### 3.3 Identification of differential expressed mRNAs in platelets between localized and metastatic cancer patients

**FIGURE 3:**
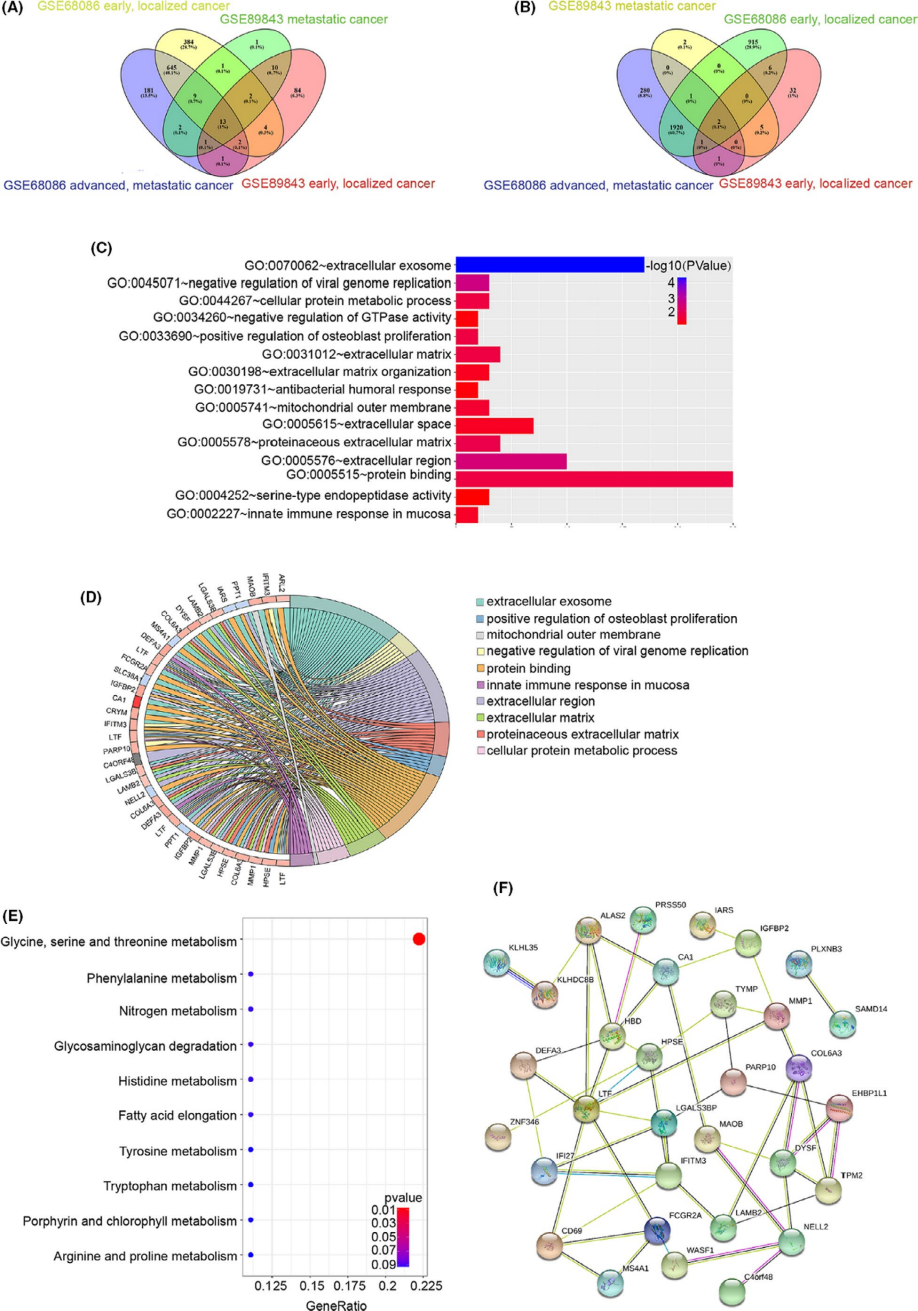
Analysis of the spliced RNA repertoire of TEPs from pan‐cancer patients at different stages. (A, B) Identification of DEGs in the four datasets (GSE68086 early pan‐cancer and metastatic pan‐cancer, GSE89843 early NSCLC cancer, and metastatic NSCLC cancer) via Venn diagrams software. (A) Up‐regulated differential expressed TEPs mRNAs. (B) Down‐regulated differential expressed TEPs mRNAs. Different colors represent different datasets. (C, D) GO analyses of the DEGs according to their biological process, cellular component, and molecular function. GO, gene ontology. (E) KEGG pathway enrichment analysis. Dot size represents the number of genes in each KEGG pathway; *p*‐value: Red < purple < blue. KEGG, Kyoto Encyclopedia of Genes and Genomes. (F) Protein‐protein interaction network of DEGs visualized through String datasets

To investigate the differentially expressed mRNAs in platelets between localized or metastatic cancer and healthy donors, we analyzed the microarray data of GSE68068 and GSE89843. Using fold change (FC) ≥ 1 as the cut‐off criterion, we extracted 3905 (1060 up‐regulated and 2845 down‐regulated) versus 164 (117 up‐regulated and 47 down‐regulated) DEGs from localized tumor educated platelets as compared to platelet samples of healthy controls, and 3059 (854 up‐regulated and 2205 down‐regulated) versus 49 (39 up‐regulated and 10 down‐regulated) DEGs from metastatic tumor educated platelets in the two datasets, respectively (Figure 1B,C, Figure 2B,C, Tables [Link], [Link], [Link], [Link]). To further investigate the key differential genes between localized tumor‐educated platelets and metastatic tumor‐educated platelets, we integrated four groups of DEGs mentioned above to further take the intersection. Using the available Venn website, 20 common DEGs were identified and 74 DEGs were found only in the localized tumor‐educated platelets, whereas 13 DEGs in the metastatic tumor‐educated platelets (Figure S3, Table S5). In addition, to further screen out the consistent altered DEGs in the localized or/and metastatic tumor‐educated platelets, we integrated the four up‐regulated/down‐regulated groups to take the intersection. We extracted 13 common DEGs, eight DEGs mainly found in tumor‐educated platelets, and 12 DEGs in the metastatic tumor‐educated platelets in the four up‐regulated groups. Moreover, we also found two common DEGs, seven DEGs mainly located in tumor‐educated platelets, and one DEGs in the metastatic tumor‐educated platelets in the four down‐regulated groups (Figure 3A,B, Table 1). DEGs mainly located in localized tumor educated platelets (PLXNB3, SAMD14, ALAS2, C4orf48, PARP10, EHBP1L1, DYSF, SSBP4, LRRC75A, CD69, RSL24D1, ZNF667, PPT1, IARS, and HERC3), DEGs mainly located in the metastatic tumor educated platelets (FCGR2A, KLHDC8B, DEFA3, IGFBP2, MAOB, ZNF346, ARL2, MMP1, KLHL35, CA1, RP11‐525A16.4, CTD‐2509G16.2, and MS4A1) and those found in both localized and metastatic tumor educated platelets (HPSE, IFI27, LGALS3BP, CRYM, WASF1, HBD, COL6A3, PRSS50, LAMB2, LTF, TPM2, TYMP, NELL2, SLC38A1, and IFITM3) were selected for further analysis.

**TABLE 1:** Consistent altered DEGs in the localized or/and metastatic tumor educated platelets

Types	Down	Up
Early/localized cancer	LRRC75A‐AS1	PLXNB3
	CD69	SAMD14
	RSL24D1	ALAS2
	ZNF667‐AS1	C4orf48
	PPT1	PARP10
	IARS	EHBP1L1
	HERC3	DYSF
		SSBP4
Common between early and advanced cancer	NELL2	HBD
	SLC38A1	IFITM3
		CRYM
		IFI27
		HPSE
		WASF1
		LGALS3BP
		TYMP
		COL6A3
		LAMB2
		TPM2
		LTF
		PRSS50
Advanced cancer	MS4A1	FCGR2A
		KLHDC8B
		DEFA3
		IGFBP2
		MAOB
		ZNF346
		ARL2
		MMP1
		KLHL35
		CA1
		RP11‐525A16.4
		CTD‐2509G16.2

### 3.4 Molecular concepts significantly enriched in tumor educated platelets

To explore the underlying mechanism and signaling pathways of these enriched DEGs, DAVID, and KEGG were performed to acquire functional and pathway enrichment analysis. GO (gene ontology) analysis was employed to functionally annotate the differentially expressed platelet RNAs. The most significant output of GO analysis was related to protein binding, extracellular matrix, cellular protein metabolic process, mitochondrial outer membrane, and innate immune response in the mucosa (Figure 3C,D). For biological processes, 43 DEGs were enriched in negative regulation of viral genome replication, positive regulation of osteoblast proliferation, cellular protein metabolic process, innate immune response in the mucosa, extracellular matrix organization, negative regulation of GTPase activity, and antibacterial humoral response. In addition, molecular functions were involved in protein binding and serine‐type endopeptidase activity.

Cell components showed that DEGs were enriched in extracellular exosome, extracellular region, proteinaceous extracellular matrix, extracellular matrix, mitochondrial outer membrane, and extracellular space (Figure 3D, Figure S4, Table S6). By using KEGG analysis, we found these 43 DEGs were significantly enriched in metabolic processes, mostly in glycine, serine, and threonine metabolism (Figure 3E, Table S7). Besides, the PPI network consisted of 41 nodes and 51 edges with a confidence score of ≥0.15. The correlated degree scores constructed with Cytoscape MCODE were 3.826, 3.684, 3.667, 2.8, 2.769, and 2.75, indicating that the platelet‐derived DEGs interacted with each other (Figure 3F).

### 3.5 Diagnostic value of RSL24D1 for early, localized cancer

**FIGURE 4:**
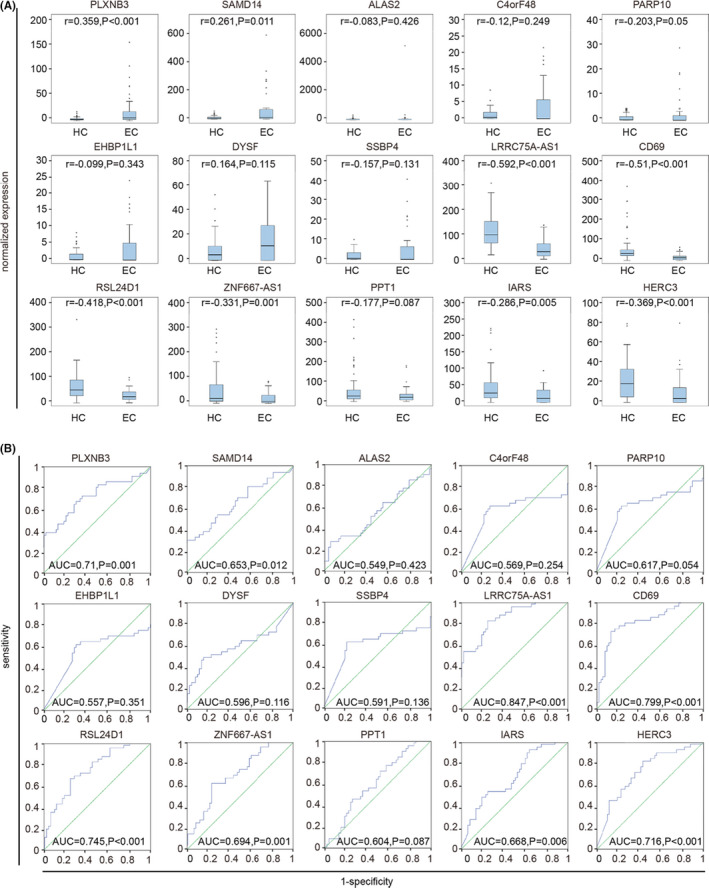
Diagnostic value of TEPs DEGs for early, localized pan‐cancer based on the datasets GSE68086. (A) Correlation analysis between expression levels of 15 TEPS DEGs and two groups, including healthy control groups and early cancer groups. HC, healthy control; EC, early cancer; The 15 TEPS DEGs are PLXNB3, SAMD14, ALAS2, C4orf48, PARP10, EHBP1L1, DYSF, SSBP4, LRRC75A, CD69, RSL24D1, ZNF667, PPT1, IARS, and HERC3, respectively. (B) ROC analysis of sensitivity and specificity of the above 15 TEPS DEGs signature in predicting the diagnosis of early pan‐cancer patients

To further investigate the signature of these TEPs DEGs, we primarily checked whether DEGs had their expression‐level changes correlated with the early, localized pan‐cancer. By using Spearman correlation coefficient, we demonstrated PLXNB3 (*r* = 0.359, *p* < 0.001), SAMD14 (*r* = 0.261, *p* = 0.011), LRRC75A (*r* = −0.592, *p* < 0.001), CD69 (*r* = −0.51, *p* < 0.001), RSL24D1 (*r* = −0.418, *p* < 0.001), ZNF667 (*r* = −0.331, *p* = 0.001), IARS (*r* = −0.286, *p* = 0.005), and HERC3 (*r* = −0.369, *p* < 0.001) were positively or negatively correlated with the early pan‐cancer based on the data from GSE68086 (Figure 4A), whereas PLXNB3 (*r* = 0.134, *p* = 0.022), SAMD14 (*r* = 0.291, *p* < 0.001), ALAS2 (*r* = 0.294, *p* < 0.001), C4orf48 (*r* = 0.249, *p* < 0.001), PARP10 (*r* = 0.192, *p* = 0.001), EHBP1L1 (*r* = 0.303, *p* < 0.001), SSBP4 (*r* = 0.212, *p* < 0.001), LRRC75A (*r* = −0.291, *p* < 0.001), CD69 (*r* = −0.191, *p* = 0.001), RSL24D1 (*r* = −0.296, *p* < 0.001), ZNF667 (*r* = −0.242, *p* < 0.001), PPT1 (*r* = −0.175, *p* = 0.003), IARS (*r* = −0.25, *p* < 0.001), and HERC3 (*r* = −0.26, *p* < 0.001) were positively or negatively correlated with the early NSCLC cancer based on the data from GSE89843 (Figure S5A). In addition, to further explore the diagnostic value of these TEPs DEGs, ROC analysis was carried out. Diagnostic analysis results showed that PLXNB3 (AUC = 0.71, *p* = 0.001), LRRC75A‐AS1 (AUC = 0.847, *p* < 0.001), CD69 (AUC = 0.799, *p* < 0.001), RSL24D1 (AUC = 0.745, *p* < 0.001), and HERC3 (AUC = 0.716, *p* < 0.001) had a diagnostic value based on the data from GSE68086 (Figure 4B), whereas SAMD14 (AUC = 0.712, *p* < 0.001), ALAS2 (AUC = 0.714, *p* < 0.001), EHBP1L1 (AUC = 0.72, *p* < 0.001), LRRC75A‐AS1 (AUC = 0.712, *p* < 0.001) and RSL24D1 (AUC = 0.715, *p* < 0.001) had a diagnostic value based on the data from GSE89843 (Figure S5B). Taking the results from two datasets into intersection, we found that RSL24D1 was negatively correlated with the early, localized cancer as compared to healthy controls and had a diagnostic value for early, localized cancer with a sensitivity of 71.8%, and a specificity of 64.3%.

### 3.6 Association of the TEPs DEGs signature with a cancer diagnosis and staging

**FIGURE 5:**
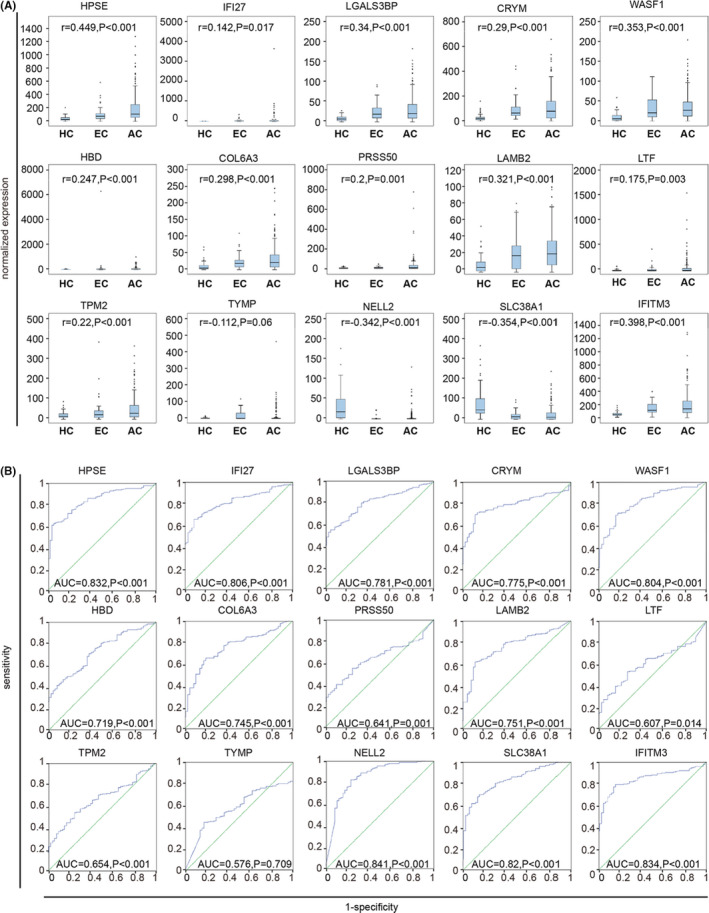
Identification of 15 TEPS DEGs signatures for pan‐cancer diagnosis based on the datasets GSE68086. (A) Correlation analysis between expression levels of 15 TEPS DEGs and three groups, including healthy control groups, early cancer groups, and advanced cancer groups. HC, healthy control; EC, early cancer; AC, advanced cancer. The 15 TEPS DEGs are HPSE, IFI27, LGALS3BP, CRYM, WASF1, HBD, COL6A3, PRSS50, LAMB2, LTF, TPM2, TYMP, NELL2, SLC38A1, and IFITM3, respectively. (B) ROC analysis of sensitivity and specificity of the above 15 TEPS DEGs signature in predicting the diagnosis of pan‐cancer patients

HPSE, IFI27, LGALS3BP, CRYM, WASF1, HBD, COL6A3, PRSS50, LAMB2, LTF, TPM2, TYMP, NELL2, SLC38A1, and IFITM3 were differentially expressed in both localized and metastatic cancer educated platelets as compared to the platelets of healthy controls. By using the Spearman correlation coefficient, we found that in addition to TYMP, the expression levels of the remaining 14 TEPs DEGs were positively or negatively related to the pan‐cancer stage based on the data from GSE68086 (Figure 5A), showing an upward or downward trend in the expression of TEPs DEGs from early to more advanced stages. In addition, 15 TEPs DEGs were positively or negatively correlated with the stage of NSCLC based on the data from GSE89843 (Figure S6A). Additionally, diagnostic analysis results indicated that HPSE, IFI27, LGALS3BP, CRYM, HBD, COL6A3, LAMB2, and IFITM3 (AUC > 0.7, *p* < 0.001)^30^ had predictive validation value for pan‐cancer based on the data from GSE68086 and GSE89843 (Figure 5B, Figure S6B). Taking both correlation and diagnostic value into consideration, we verified that HPSE, IFI27, LGALS3BP, CRYM, HBD, COL6A3, LAMB2, and IFITM3 were positively related to pan‐cancer stage and had potential value for pan‐cancer diagnosis and staging with a sensitivity of 60.9%, 59.1%, 56.5%, 57.8%, 54.3%, 55.2%, 55.2%, and 60.9%, and a specificity of 94.5%, 90.9%, 87.3%, 89.1%, 72.7%, 85.5%, 89.1%, and 94.5%, respectively.

### 3.7 Diagnostic value of ARL2, FCGR2A, and KLHDC8B for advanced, metastatic cancer

**FIGURE 6:**
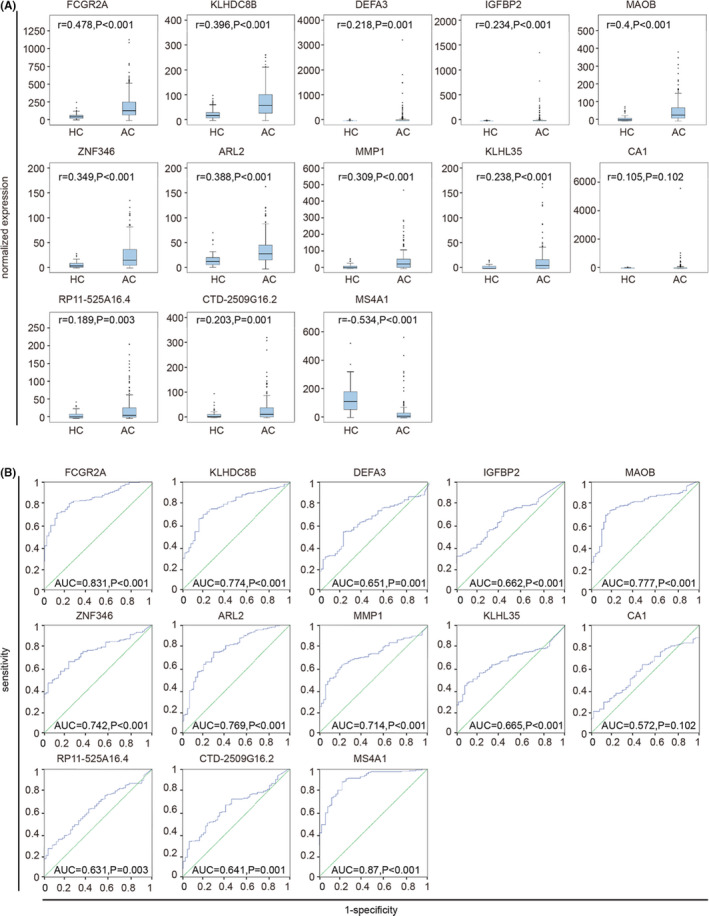
Diagnostic value of 13 TEPs DEGs for advanced, metastatic pan‐cancer based on the datasets GSE68086. (A) Correlation analysis between expression levels of 13 TEPS DEGs and two groups, including healthy control groups and advanced cancer groups. HC, healthy control; AC, advanced cancer. The 13 TEPS DEGs are FCGR2A, KLHDC8B, DEFA3, IGFBP2, MAOB, ZNF346, ARL2, MMP1, KLHL35, CA1, RP11‐525A16.4, CTD‐2509G16.2, and MS4A1, respectively. (B) ROC analysis of sensitivity and specificity of the above 15 TEPS DEGs signature for predicting advanced pan‐cancer

FCGR2A, KLHDC8B, DEFA3, IGFBP2, MAOB, ZNF346, ARL2, MMP1, KLHL35, CA1, RP11‐525A16.4, CTD‐2509G16.2, and MS4A1 are differentially expressed in advanced, metastatic cancers. In this study, we explored the diagnostic value of these TEPs DEGs for advanced, metastatic cancers. By using Spearman correlation coefficient, we identified that apart from CA1, the expression levels of the remaining 12 TEPs DEGs were positively or negatively correlated with advanced cancers through integrating two datasets from GSE68086 and GSE89843 (Figure 6A, Figure S7A). Besides, diagnostic analysis results showed that FCGR2A (AUC = 0.831, *p* < 0.001), KLHDC8B (AUC = 0.774, *p* < 0.001), MAOB (AUC = 0.77, *p* < 0.001), ZNF346 (AUC = 0.742, *p* < 0.001), ARL2 (AUC = 0.769, *p* < 0.001), MMP1 (AUC = 0.714, *p* < 0.001), and MS4A1 (AUC = 0.87, *p* < 0.001) had the diagnostic value based on the data from GSE68086 (Figure 6B). However, by analyzing GSE89843 data, we found that only FCGR2A (AUC = 0.705, *p* < 0.001), KLHDC8B (AUC = 0.707, *p* < 0.001), IGFBP2 (AUC = 0.711, *p* < 0.001), and ARL2 (AUC = 0.791, *p* < 0.001) had the diagnostic value for metastatic NSCLC cancer (Figure S7B). Taking these results into intersection, we discovered that ARL2, FCGR2A, and KLHDC8B were negatively correlated with the advanced, metastatic pan‐cancer in comparison with healthy controls and they were essential for advanced, metastatic cancers diagnosis with a sensitivity of 59.2%, 61.8%, and 59.7%, and a specificity of 80%, 89.1%, and 83.6%, respectively.

### 3.8 Validation of selected DEGs by qRT‐PCR

**FIGURE 7:**
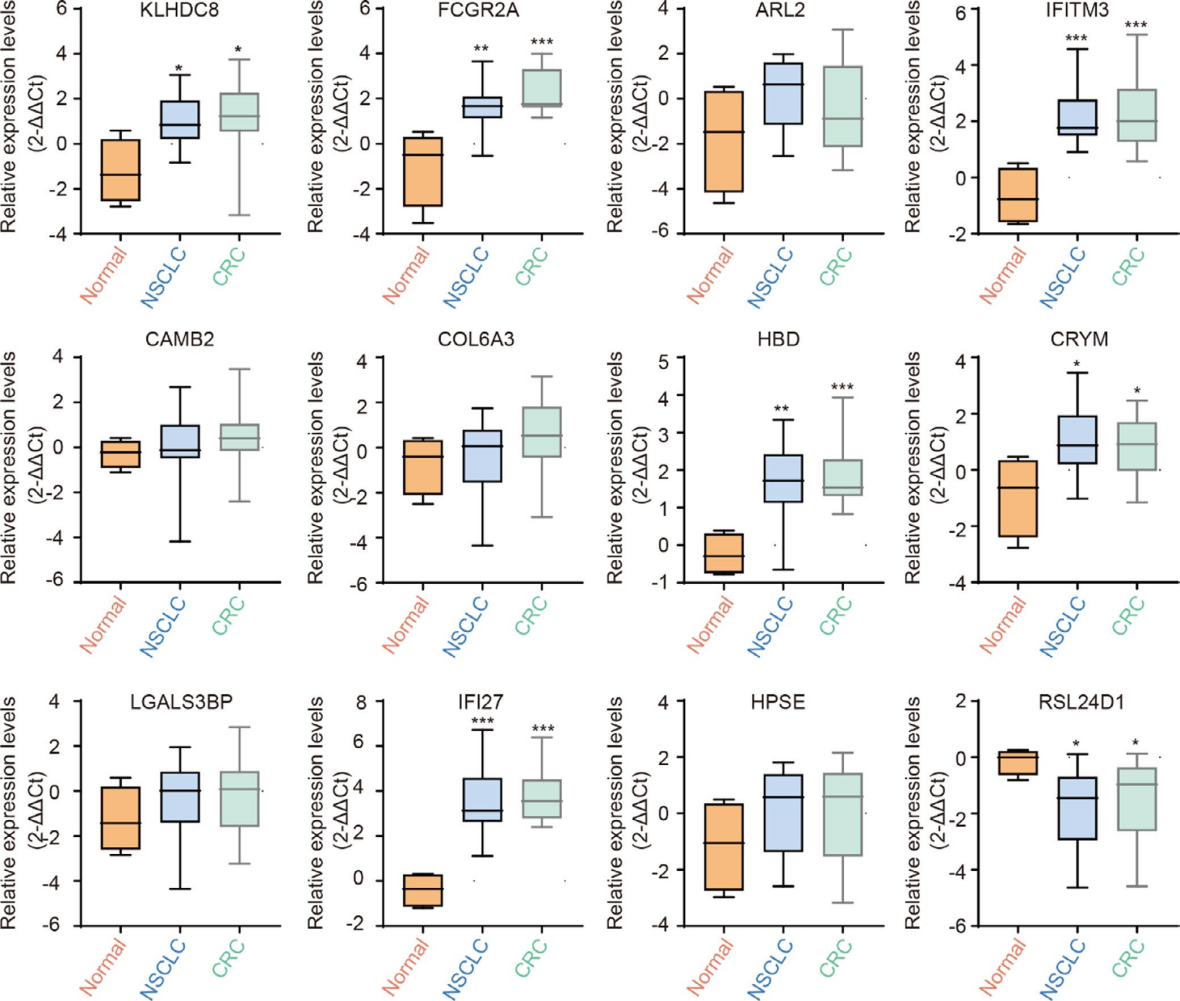
Relative expression level of 12 selected DEGs. Relative expression level of KLHDC8B, FCGR2A, ARL2, IFITM3, LAMB2, COL6A3, HBD, CRYM, LGALS3BP, IFI27, HPSE and RSL24D1. NSCLC: non‐small cell lung carcinoma; CRC: colorectal cancer. *p < 0.05, **p < 0.01, and ***p < 0.001

To confirm and raise the credibility of 12 DEGs originating from the public datasets, we collected and washed the platelets from NSCLC, CRC cancer patients, and healthy volunteers to further test the expression of DEGs. The relative expression levels of selected DEGs were detected using qRT‐PCR. Among the 12 selected DEGs, the relative expression of RSL24D1 was down‐regulated in cancer patients' platelets, while IFI27, CRYM, HBD, IFITM3, FCGR2A, and KLHDC8B were up‐regulated in cancer patients' platelets, which was consistent with the results analyzed by using bioinformatics (Figure 7).

## 4 DISCUSSION

In this study, we downloaded the next‐generation sequencing datasets, GSE68086 (285 samples) and GSE89843 (636 samples), from GEO (https://www.ncbi.nlm.nih.gov/geo/). We then explored the mechanisms of changes in TEPs RNA profiles by using bioinformatics analysis, correlation analysis, diagnostic analysis, and qRT‐PCR. Seven TEPs mRNAs were discovered in our study: RSL24D1, IFI27, CRYM, HBD, IFITM3, FCGR2A, and KLHDC8B in TEPs, which are mainly enriched in protein binding, extracellular matrix, and metabolic process, and may be used for cancer diagnosis.

Blood samples for liquid biopsy include circulating tumor cells (CTCs), cell‐free nucleic acids, exosomes (DNA, RNA, miRNA, proteins), and TEPs.^31^ Liquid biopsy has been shown to be useful for the early detection of cancers compared to CTCs, cell‐free nucleic acids, and exosomes found at lower levels at the early stage.^31^, ^32^ Platelet‐associated indicators and platelet counts have been shown to be associated with the prognosis of resectable lung and colorectal cancers.^33^, ^34^, ^35^ Moreover, Best et al^9^ used NGS to examine 283 blood platelet samples isolated from healthy controls and patients with cancer and found that RNA profiles of TEPs could be used to diagnose cancer patients with 96% accuracy. Recent studies have demonstrated that TIMP1 and TGA2B mRNA in tumor‐educated platelets are good diagnostic biomarkers for colorectal cancer and lung cancer, respectively.^21^, ^22^ To sum up, these studies suggest that TEPs mRNAs may be used as liquid‐biopsy biomarkers for cancer diagnosis. Nevertheless, it remains unclear how much TEP mRNAs are required for accurate diagnosis and which genes are included in TEP mRNAs.

In this study, 7 TEPs mRNAs were verified, including RSL24D1, IFI27, CRYM, HBD, IFITM3, FCGR2A, and KLHDC8B, which may be used for cancer detection. RSL24D (ribosomal L24 domain containing 1, also known as C15orf15, RPL24L, or My024) encodes ribosome biogenesis protein RLP24 involved in the biogenesis of the 60S ribosomal subunit, ensuring the docking of GTPBP4/NOG1 to pre‐60S particles. RSL24D1 is related to hypercholesterolemia and children's chronic kidney disease (CKD).^36^, ^37^ A recent genome‐wide methylation profile analysis indicated that the change of RSL24D1 was associated with advanced‐stage NSCLC.^38^ Our study found that RSL24D1 in TEPs was negatively associated with cancers at an early stage, including breast cancer, lung cancer, CRC, PAAD, and HBC, compared to healthy controls. In addition, we also demonstrated the diagnostic value of RSL24D1 for early pan‐cancer with a sensitivity of 71.8% and a specificity of 64.3%.

FCGR2A and KLHDC8B in TEPs were positively related to metastatic cancers in comparison with healthy controls and had potential diagnostic significance for metastatic cancers with a sensitivity of 61.8%, 59.7%, and a specificity of 89.1%, 83.6%, respectively. Previous studies reported that FCGR2A regulates cancer growth, cancer invasion and has an important role in tumor recurrence.^39^, ^40^ On the other hand, the role of KLHDC8B in tumors is not clear.

IFI27, CRYM, HBD, and IFITM3 in TEPs were positively related to the pan‐cancer stage and essential for diagnosing cancers with a sensitivity of 59.1%, 57.8%, 54.3%, and 60.9%, and a specificity of 90.9%, 89.1%, 72.7%, and 94.5%, respectively. Ketimine reductase mu‐crystallin and hemoglobin subunit delta encoded by CRYM and HBD, respectively, did not seem to be associated with tumor progression. IFI27 (interferon alpha‐inducible protein 27) is involved in tumor apoptosis signaling pathways, including type‐I interferon‐induced apoptosis and TNFSF10‐induced apoptosis,^41^, ^42^, ^43^ and regulates the innate immune response.^44^, ^45^ The up‐regulation of IFI27 participates in the invasion and proliferation of oral squamous cell carcinoma, pancreatic ductal adenocarcinoma, and breast cancer.^46^, ^47^, ^48^ IFITM3P has a role in promoting both blood and solid tumors through different signaling pathways.^49^, ^50^, ^51^

According to the GO term analysis and KEGG pathway analysis, we found that TEPs mRNAs were correlated with protein binding, extracellular matrix, cellular protein metabolic process, mitochondrial outer membrane, and innate immune response in the mucosa, as well as enriched metabolic process, mostly glycine, serine, and threonine metabolism. As one of the ten cancer hallmarks, metabolic abnormalities are mutually causal with tumor tumorigenesis.^52^ Tumor blood metastasis can be divided into three stages: (a) translocation of vascular endothelial cells from tumor cells through the tumor tissue from the primary site; (b) tumor cells rolling with the blood; (c) implantation of tumor cells in the metastatic site. Multiple cell adhesion molecules, extracellular matrix, and other blood cells are involved in the metastatic tumor process.^53^ Recently, platelets have been found to have a critical role in promoting tumorigenesis during metastasis. The following mechanisms have been proposed: (1) platelets can be activated by tumor cells in the blood vessels, after which they aggregate around tumor cells to form a tumor thrombus, thereby protecting the tumor cells from the immune system attack; (2) meanwhile, platelets can adhere to endothelial cells and tumor cells through surface adhesion molecules, such as p‐selectin, which can act as a bridge between tumor cells and endothelial cells, helping tumor cells to adhere to the vascular endothelial cells at the metastatic site; (3) platelets can secrete a variety of biological factors, then promote tumor growth and angiogenesis of tumor tissue.^16^, ^54^ Consequently, we believe alternative TEPs mRNAs, including RSL24D1, IFI27, CRYM, HBD, IFITM3, FCGR2A, and KLHDC8B mRNA, can potentially serve as non‐invasive biomarkers for diagnosing cancers and can even predict the prognosis of pan‐cancer. Yet, more scientific research and evidence are needed to further verify this conclusion.

## CONFLICT OF INTEREST

The authors declare no conflict of interest.

## Supporting information

Fig S1‐S7

Table S1

Table S2

Table S3

Table S4

Table S5

Table S6

Table S7

Table S8

## Data Availability

All data are available at reasonable request to the corresponding author.
