# Supplementary material for: Identification of seven tumor‐educated platelets RNAs for cancer diagnosis
Source: J Clin Lab Anal. 2021 May 6;35(6):e23791. doi: 10.1002/jcla.23791 (PMC8183939; doi:10.1002/jcla.23791)
Supplement: Supplementary file 1 — Fig S1‐S7 [file JCLA-35-e23791-s009.docx]

**Supplementary Figures**


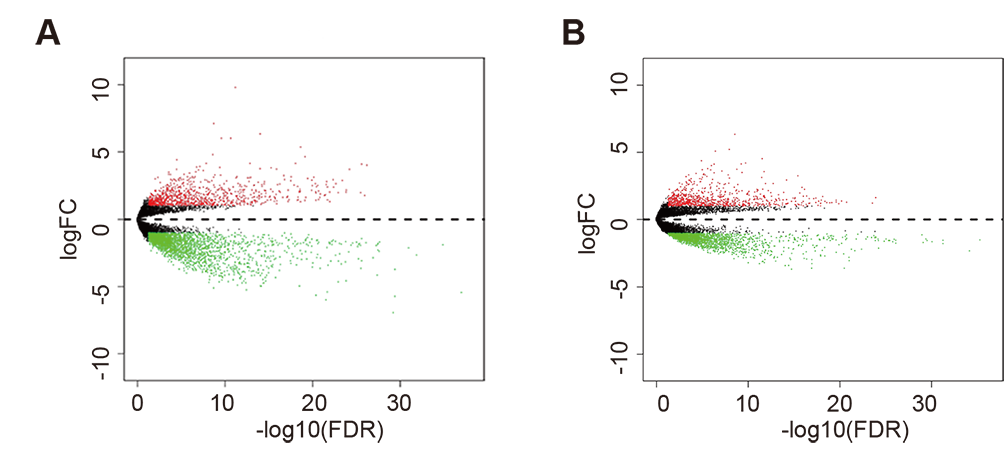


**Figure S1. A,** Volcano plot of 3905 TEPs DEGs from localized pan-cancer patients compared to platelets from healthy controls based on the datasets GSE68086. **B,** Volcano plot of 3059 TEPs DEGs from metastatic pan-cancer patients compared to platelets from healthy controls based on the datasets GSE68086. Red, FC > 1 and adj P-value < 0.05; green, FC < 1 and adj P-value < 0.05. FC, fold change.


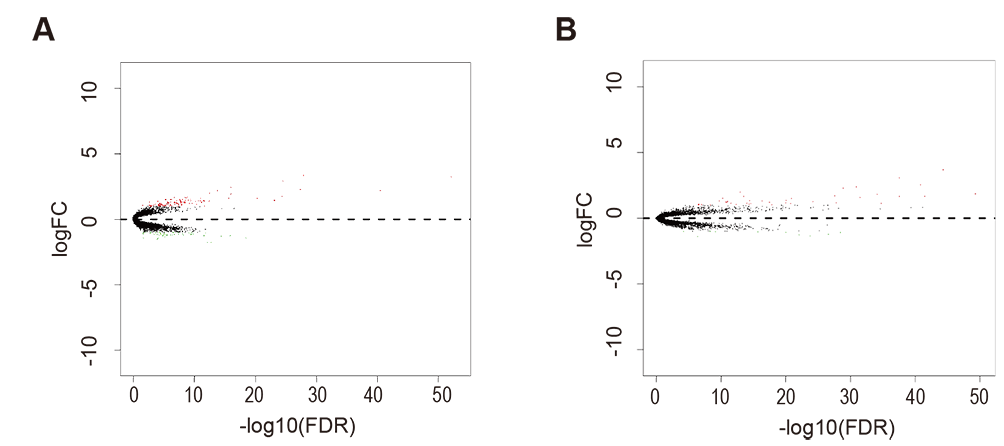


**Figure S2. A,** Volcano plot of 164 TEPs DEGs from localized NSCLC cancer patients compared to platelets from healthy controls based on the datasets GSE89843. **B,** Volcano plot of 49 TEPs DEGs from metastatic NSCLC cancer patients compared to platelets from healthy controls based on the datasets GSE89843. Red, FC > 1 and adj P-value < 0.05; green, FC <1 and adj P-value < 0.05. FC, fold change.


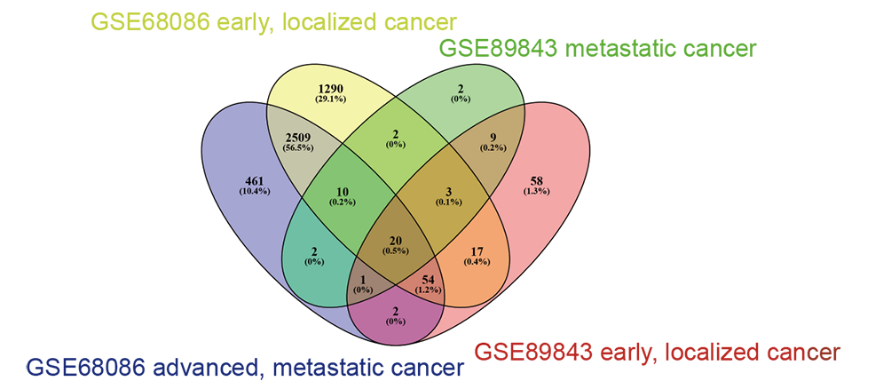


**Figure S3.** Identification of commonly altered DEGs in the four datasets (GSE68086 early pan-cancer and metastatic pan-cancer, GSE89843 early NSCLC cancer, and metastatic NSCLC cancer) via Venn diagrams software.


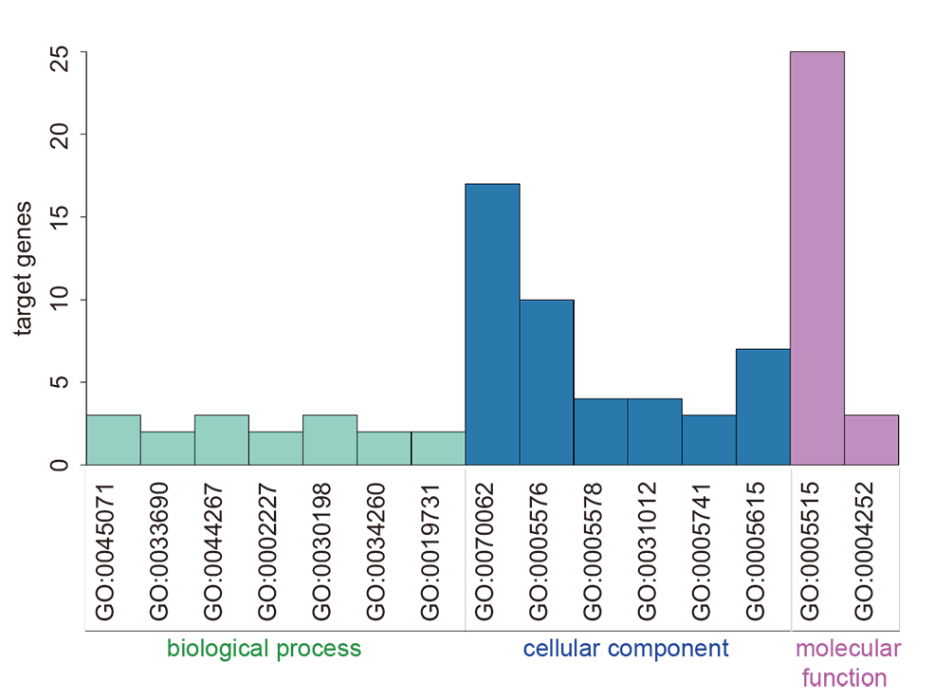


**Figure S4.** GO analyses of the DEGs according to their biological process, cellular component, and molecular function.


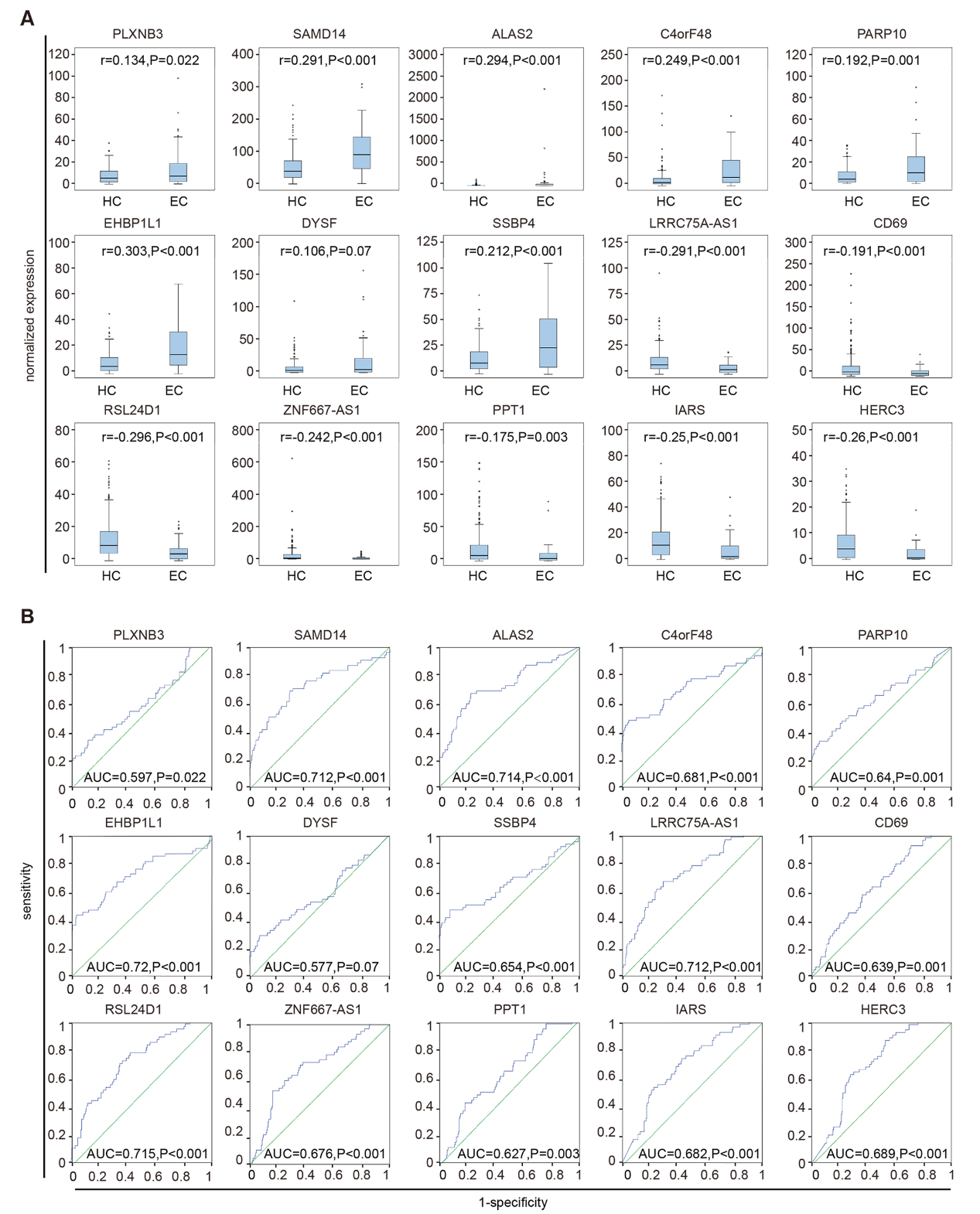


**Figure S5.** **A,** Correlation analysis between expression levels of 15 TEPS DEGs and two groups, including healthy control groups and early NSCLC cancer groups based on the datasets GSE89843. HC, healthy control; EC, early cancer. The 15 TEPS DEGs are PLXNB3, SAMD14, ALAS2, C4orf48, PARP10, EHBP1L1, DYSF, SSBP4, LRRC75A, CD69, RSL24D1, ZNF667, PPT1, IARS, and HERC3, respectively. **B,** ROC analysis of sensitivity and specificity of the above 15 TEPS DEGs signature in predicting the diagnosis of early NSCLC cancer patients.


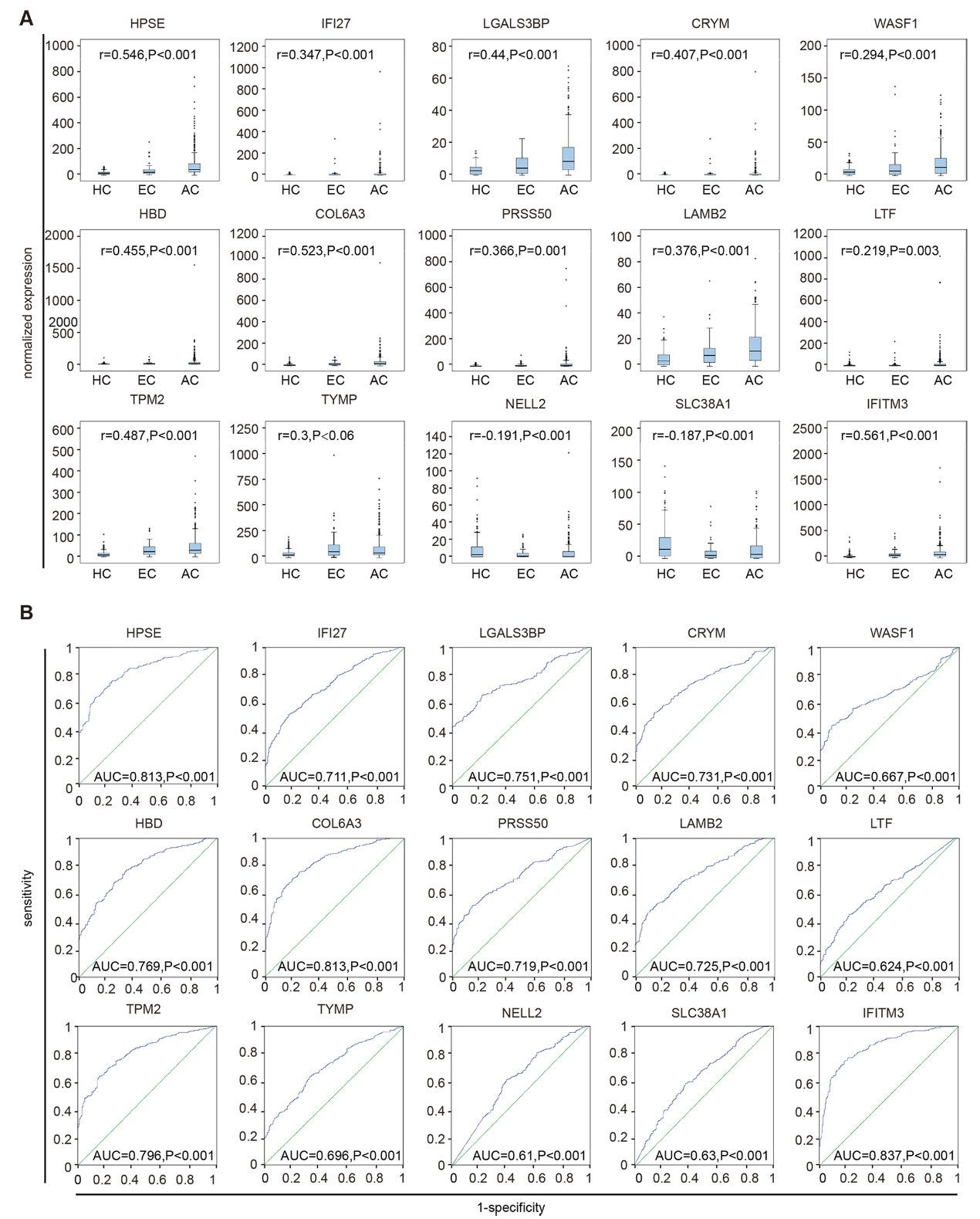


**Figure S6.** **A,** Correlation analysis between expression levels of 15 TEPS DEGs and three groups, including healthy control groups, early cancer groups, and advanced cancer groups based on the datasets GSE89843. HC, healthy control; EC, early cancer; AC, advanced cancer. The 15 TEPS DEGs are HPSE, IFI27, LGALS3BP, CRYM, WASF1, HBD, COL6A3, PRSS50, LAMB2, LTF, TPM2, TYMP, NELL2, SLC38A1, and IFITM3, respectively**. B,** ROC analysis of sensitivity and specificity of the above 15 TEPS DEGs signature in predicting the diagnosis of NSCLC cancer patients.


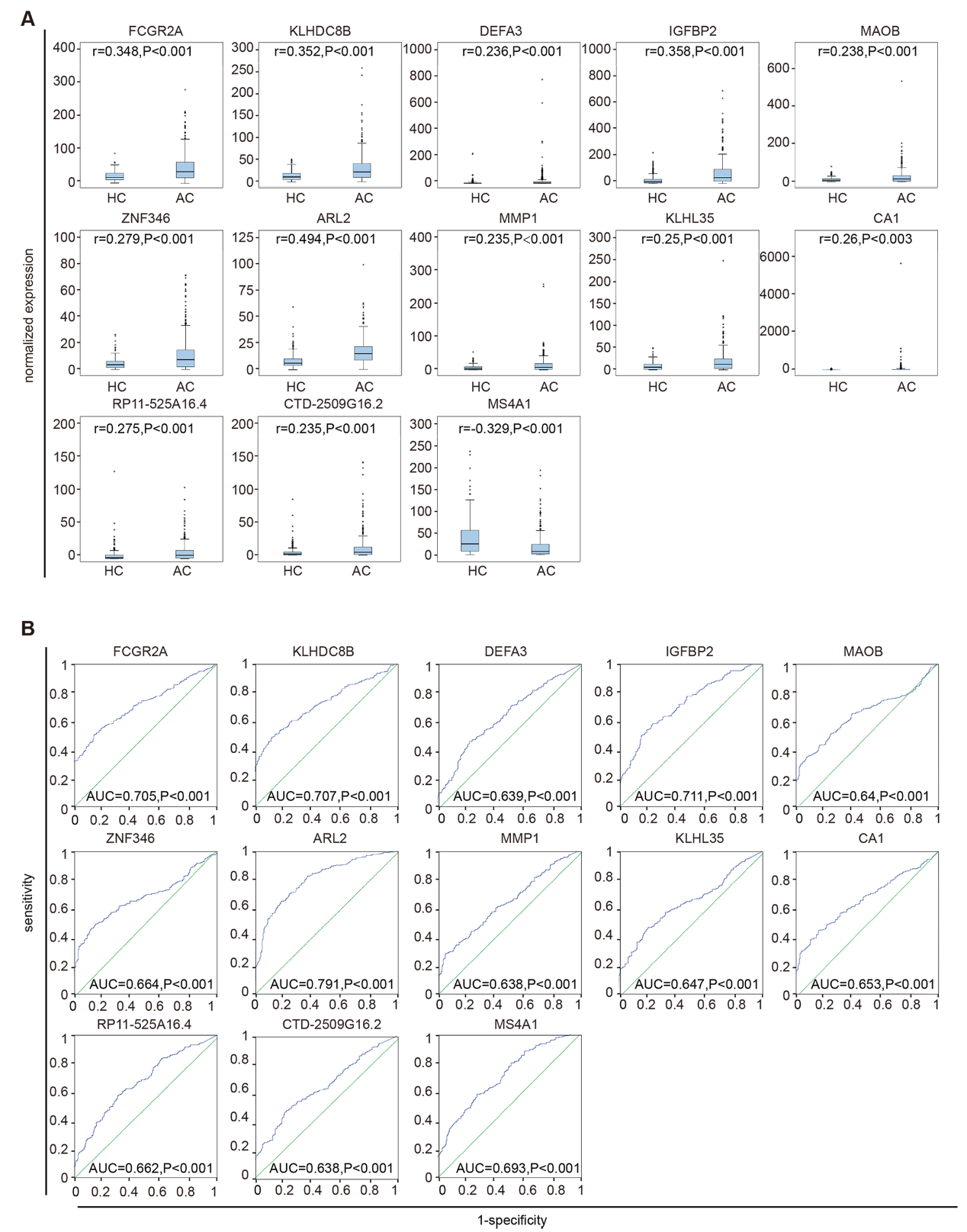


**Figure S7.** **A,** Correlation analysis between expression levels of 13 TEPS DEGs and two groups, including healthy control groups and advanced cancer groups based on the datasets GSE89843. HC, healthy control; AC, advanced cancer. The 13 TEPS DEGs are FCGR2A, KLHDC8B, DEFA3, IGFBP2, MAOB, ZNF346, ARL2, MMP1, KLHL35, CA1, RP11-525A16.4, CTD-2509G16.2, and MS4A1, respectively. **B,** ROC analysis of sensitivity and specificity of the above 15 TEPS DEGs signature in predicting the diagnosis of advanced NSCLC cancer patients.
