# Supplementary material for: Identification of seven tumor‐educated platelets RNAs for cancer diagnosis
Source: J Clin Lab Anal. 2021 May 6;35(6):e23791. doi: 10.1002/jcla.23791 (PMC8183939; doi:10.1002/jcla.23791)
Supplement: Supplementary file 9 — Table S8 [file JCLA-35-e23791-s001.docx]

**Table S8. Clinical characteristics of volunteers**

| Donors | Age | Sex | Classificatiion |
| --- | --- | --- | --- |
| 1 | 52 | Male | NSCLC |
| 2 | 46 | Female | NSCLC |
| 3 | 48 | Female | NSCLC |
| 4 | 75 | Male | NSCLC |
| 5 | 44 | Female | NSCLC |
| 6 | 64 | Female | NSCLC |
| 7 | 42 | Male | NSCLC |
| 8 | 40 | Female | NSCLC |
| 9 | 44 | Female | NSCLC |
| 10 | 64 | Male | NSCLC |
| 11 | 65 | Male | NSCLC |
| 12 | 71 | Male | NSCLC |
| 13 | 77 | Female | NSCLC |
| 14 | 73 | Male | NSCLC |
| 15 | 40 | Male | NSCLC |
| 16 | 69 | Female | NSCLC |
| 17 | 32 | Female | CRC |
| 18 | 61 | Male | CRC |
| 19 | 57 | Male | CRC |
| 20 | 64 | Female | CRC |
| 21 | 55 | Male | CRC |
| 22 | 50 | Male | CRC |
| 23 | 58 | Male | CRC |
| 24 | 61 | Female | CRC |
| 25 | 48 | Male | CRC |
| 26 | 69 | Male | CRC |
| 27 | 58 | Female | CRC |
| 28 | 74 | Male | CRC |
| 29 | 49 | Male | CRC |
| 30 | 79 | Female | CRC |
| 31 | 74 | Male | CRC |
| 32 | 31 | Female | CRC |
| 33 | 66 | Male | CRC |
| 34 | 43 | Female | CRC |
| 35 | 75 | Male | CRC |
| 36 | 24 | Male | Healthy volunteer |
| 37 | 24 | Male | Healthy volunteer |
| 38 | 24 | Male | Healthy volunteer |
| 39 | 24 | Male | Healthy volunteer |
